# Supplementary material for: Simulation-Based Gastrointestinal Endoscopy Sedations: A Novel Validation to Multidrug Pharmacodynamic Modeling
Source: Pharmaceutics. 2022 Sep 27;14(10):2056. doi: 10.3390/pharmaceutics14102056 (PMC9610933; doi:10.3390/pharmaceutics14102056)
Supplement: Supplementary file 1 [file pharmaceutics-14-02056-s001.zip › pharmaceutics-1901134-supplementary.pdf]

Supplementary S1. Pharmacokinetic and Pharmacodynamic Models

The study involved series of pharmacokinetic and pharmacodynamic simulations. The general approach is to calculate the pharmacokinetic effect-site drug concentrations (Ces) and use the Ces in the pharmacodynamic model to calculate drug effects. The pharmacokinetic and pharmacodynamic models presented here are published models. Model selection and model fit are not performed as a part of the study. The models are described below.

Pharmacokinetic simulation

Effect-site concentrations (Ces) are calculated with TIVA trainer (Version 9.1, Build 6, EuroSIVA). The simulation is based on a female who is 60-year-old, 170 cm and 65kg.[17, 21] The Maitre model[22] was used for alfentanil, Zomorodi model[23] for midazolam, Shafer model[24] for fentanyl and Schnider model[25] for propofol. Ces are calculated for 60 minutes at 5-second intervals for better temporal resolution. Opioids are converted to alfentanil equivalents based on the potency ratio fentanyl:alfentanil:remifentanil = 1:0.0625:1.2[1]. This process is necessary to compare different opioids and is a common technique used in anesthesia pharmacodynamic studies[26, 27].

The Pharmacodynamic model

The Non-linear Mixed Effect with Zero amounts (NLMAZ) three-drug response surface model is used as our simulation model.[9] The general form of the model follows the sigmoid-E_{max} curve (Equation 2)：

$$E = \frac{(E_{max} - E_0) \times \left(\frac{U}{U_{50}}\right)^m}{1 + \left(\frac{U}{U_{50}}\right)^m} + E_0 \qquad [2]$$

E is the effect, defined as the probability of LOR. Modified OAA/S (MOAA/S) < 2 is used to represent LOR. E_{max} is the maximal drug effect possible and E_0 is the baseline probability when no drugs are present. U_{50} and is the drug concentrations required for the drug *U* to take 50% maximal effect, that is, to achieve 50% chance of LOR. Parameter *m* is the slope factor that determines the steepness of the LOR change[9]. U can be interpreted as a new drug and is the sum of the normalized potency of midazolam, alfentanil and propofol (equation 3 and 4):

$$\mathrm{U}_m = \frac{C_m}{C_{50m}} \qquad \mathrm{U}_a = \frac{C_a}{C_{50a}} \qquad \mathrm{U}_p = \frac{C_p}{C_{50p}} \qquad [3]$$

$$U = \mathrm{U}_m + \mathrm{U}_a + \mathrm{U}_p \qquad [4]$$

The variables C_m, C_a and C_p refer to the calculated Ce of midazolam, alfentanil, and propofol respectively. For consistency throughout the article, the subscripts m, a, p will refer as midazolam, alfentanil and propofol respectively. C_{50} is defined as the concentration of drug required to provide half maximal effect. For the model to scale correctly, we have to define:

$$1 = \mathrm{x} + \mathrm{y} + \mathrm{z} = \frac{\mathrm{U}_m}{U} + \frac{\mathrm{U}_a}{U} + \frac{\mathrm{U}_p}{U} \qquad [5]$$

$$\mathrm{x} = \frac{\mathrm{U}_m}{U};\ \ \mathrm{y} = \frac{\mathrm{U}_a}{U};\ \ \mathrm{z} = \frac{\mathrm{U}_p}{U} \qquad [6]$$

Where x, y and z are the drug fractions of midazolam, alfentanil and propofol. The unknown parameters (P) are defined using the full cubic form of the canonical polynomial, as in equation 7:

$$\mathrm{P} = \sum \alpha_i x_i + \sum \beta_{ij} x_i x_j + \sum \gamma_{ij} x_i x_j \left(x_i - x_j\right) + \sum \delta_{ijk} x_i x_j x_k \qquad [7]$$

This can be expanded into:

$$\mathrm{P} = \alpha_1 x + \alpha_2 y + \alpha_3 z + \beta_{12} xy + \beta_{13} xz + \beta_{23} yz + \gamma_{12} xy(x - y) + \gamma_{13} xz(x - z) + \gamma_{23} yz(y - z) + \delta_{123} xyz \qquad [8]$$

The Greek letter constants are referred to as the vector constants. Equation 8 is a generalized form of the parameters n and U_{50}, where they substitute P and each has their designated vector constants:

$$n = \alpha_{n,m}x + \alpha_{n,a}y + \alpha_{n,p}z + \beta_{n,ma}xy + \beta_{n,mp}xz + \beta_{n,ap}yz + \gamma_{n,ma}xy(x-y) + \gamma_{n,mp}xz(x-z) + \gamma_{n,ap}yz(y-z) + \delta_{n,map}xyz \quad [9]$$

$$\log_{10} U_{50} = (1-x)(1-y)(1-z)\big[\alpha_{U,m}x + \alpha_{U,a}y + \alpha_{U,p}z + \beta_{U,ma}xy + \beta_{U,mp}xz + \beta_{U,ap}yz + \gamma_{U,ma}xy(x-y) + \gamma_{U,mp}xz(x-z) + \gamma_{U,ap}yz(y-z) + \delta_{U,map}xyz\big] \quad [10]$$

There are a total of 23 parameters for the MOAA/S < 2 model used for the simulation. The primary parameters are C_{50mid}=51.2 ng/mL, C_{50alf}=627.9 ng/mL and C_{50prop}=2.58 mcg/mL[9], which represent the C_{50} of midazolam, alfentanil and propofol respectively. The complete parameters are listed in Appendix Table 1.

Supplementary Table S1. Model parameters.

Parameter	Value	Parameter	Value

$C_{50,alf}$	51.2	δ_{D123}	-0.31
$C_{50,mid}$	627.9	α_{m1}	5.51
$C_{50,prop}$	2.58	α_{m2}	9.4
α_{D1}	-0.21	α_{m3}	9.3
α_{D2}	-0.26	β_{m12}	2.1
α_{D3}	-0.23	β_{m13}	-0.2
β_{D12}	-0.99	β_{m23}	9.5
β_{D13}	-0.14	γ_{m12}	-0.0075
β_{D23}	-0.3	γ_{m13}	-0.0078
γ_{D12}	-0.07	γ_{m23}	-0.0098
γ_{D13}	-0.03	δ_{m123}	-15.41
γ_{D23}	0.08		

Greek letters are the vector constants of the pharmacodynamic model.
